# Modification of egg yolk fatty acids profile by using different oil sources

**Published:** 2015-06-15

**Authors:** Mohsen Omidi, Shaban Rahimi, Mohammad Ali Karimi Torshizi

**Affiliations:** 1 *Graduate Student, Faculty of Agriculture, Tarbiat Modarres University, Tehran, Iran; *; 2 *Department of Poultry Science, Faculty of Agriculture, Tarbiat Modarres University, Tehran, Iran.*

**Keywords:** Egg enrichment, Fatty acids, Laying hen, Performance

## Abstract

The study was conducted to evaluate the effects of different dietary oil sources supplementation on laying hens’ performance and fatty acids profile of egg yolks. Seventy-two 23-week-old laying hens (Tetra-SL) divided into six experimental diets (four replicates and three birds per replication) in a completely randomized design for nine weeks. Experimental diets were included: 1) control (no oil), 2) 3.00% fish oil, 3) 3.00% olive oil, 4) 3.00% grape seed oil, 5) 3.00% canola oil, and 6) 3.00% soybean oil. The diets were similar in terms of energy and protein. Egg production, egg mass, egg weight, feed intake, feed conversion ratio and fatty acid composition of egg yolk were determined at the end of the trial. The results indicated that the performance parameters were not significantly different between treatments in the entire period (*p* > 0.05). However, fatty acids profiles of yolk were affected by experimental diets (*p* < 0.05). Fish oil significantly reduced omega-6 fatty acids and increased docosahexaenoic acid (DHA) and eicosapentaenoic acid (EPA) in egg yolk. Also canola oil increased linolenic acid content in the egg yolk. In conclusion, fish oil increased omega-3 long-chain fatty acids and decreased omega-6 to omega-3 ratio in eggs which may have beneficial effects on human health.

## Introduction

Oil and fats are usually added to the diet of poultry to enhance the energy density to produce energy-rich formulations. In order to ensure adequate levels of linoleic acid, and to improve palatability and reduce dustiness of diets, all poultry diets require a minimum of 1% added fat, regardless of other economic or nutritional considerations.^1^ It was shown that there was different constitution in terms of fatty acids (FAs) structure. Fatty acids contain carbon, oxygen and hydrogen and classified as saturated fatty acids (SFA), monounsaturated FAs (MUFA), or polyunsaturated fatty acids (PUFA). Animal fat contain especially palmitic acid as a long chain (LC) SFA except for fish oil. While vegetable oils contain high quantities of long-chain unsaturated FAs. Several studies have shown better utilization of un-saturated fats with a higher metabolizable energy compared with saturated fats. Studies with rats and broilers have shown that unsaturated vegetable oils produce lower fecal energy losses and, consequently, higher ME than animal fats.^2^^,^^3^ Also, studies with laying hens have been shown that unsaturated vegetable oils have higher energy levels than saturated animal fats. Oil supplements are added to layer hen rations to increase the absorption of the fat soluble vitamins and to enhance egg yield and egg weight.^4^

In recent years, consumer demands for more healthy foods supported the interest in modifying the FA profile of eggs. Omega-3 FAs are essential for normal growth and development and play important roles in the prevention and treatment of coronary heart disease (CRD), hyper-tension, inflammatory, autoimmune disorders and cancer.^5^ However, since consumption of fish, the richest dietary source of LC n-3 PUFA, in diets is low, intakes of LC n-3 PUFA are low and suboptimal.^6^ Researchers agree that the optimal ratio of omega-6 to omega-3 should not exceed 2:1- 4:1. The dietary imbalance in FAs (excessive omega-6 and insufficient omega-3) is an underlying cause of many chronic disease including cardiovascular disease, cancers, inflammatory diseases, autoimmune diseases and many physiological disturbances.^7^

Fish oil contains unsaturated FAs with long omega-3 chains (LC-n-3 PUFA), ecosapentaenoic acid (EPA20:5n-3) and decosahexaenoic acid (DHA22:6n-3) that improve health-related factors in humans and animals.^8^ High intakes of long-chain n-3 polyunsaturated FAs are associated with a decreased risk of cardiovascular disease.^9^^,^^10^ Many studies are directed towards the manipulation of the FA composition of broiler chicks and laying hens in order to increase n-3 PUFA content and decrease n-6/n-3 ratio in poultry meat and egg.^11^ It is possible to modify the FA profile of the yolk by changing the lipid sources of the hen diet. Nutritional manipulation of hen diets to include sources of PUFA n-3 promotes deposition of these nutrients into egg yolk.^12^

The objective of this study was to compare the effects of different oil sources on performance and FA composition of egg yolk in laying hens.

## Materials and Methods

**Animals and experimental design.** Seventy-two 23-week-old laying hens (Tetra-SL) divided randomly into six dietary treatments (four replicates and three birds per replication). Each three birds were housed in one cage (40×40×50 cm). Environmental temperature was set at 22 ˚C. A regime of 14 hr constant lighting (15 lux) and continuous ventilation were provided. All the birds were kept under uniform management conditions throughout the experimental period. Experimental diets were included: 1) Control (no oil), 2) 3.00% fish oil, 3) 3.00% olive oil, 4) 3.00% grape seed oil, 5) 3.00% canola oil, and 6) 3.00% soybean oil. Diets were formulated according to the recommendations of the National Research Council.^13^ Feed and water were provided *ad libitum* throughout the experiment. Diets were modulated as isoenergetic and iso-nitrogenous. Composition of the diets is shown in Table 1. At the end of the trial (9^th^ week), seven eggs were randomly selected from each group for determination of egg yolk FAs profile.

**Performance record. **Egg production, egg weight, feed intake, egg mass and feed conversion ratio of each pen were recorded weekly (Table 2).

**Fatty acid content. **The FA composition of the dietary oil and yolk samples was determined by gas chromatography according to the method described by Metcalfe *et al.*^14^ The FA content was determined using a gas chromatograph (Model 4600; Unicam, Cambridge, England) equipped with a BPX70 fused silica capillary column and a flame ionization detector (Unicam, Cambridge, UK). The column head pressure of the carrier gas (Helium) was 20 psi and sample volume injected was 0.2 μL. Pentadecanoic acid (Sigma-Aldrich, St. Louis, USA) was used as internal standard. The FA’s were identified by matching their retention times with those of their corresponding standards.

**Statistical analyses. **Data were analyzed in a completely randomized design, using SAS software (Version 8.0; SAS Institute, Cary, USA). Significant differences among treatments were determined according to the general linear model (GLM) procedure. Means were compared by using Duncan's multiple-range test and significance was determined when the *p*-value was less than 0.05. The following model was used:

*X*_ij_ *= μ + τ*_j_ *+ ε*_ij_

where, *X*_ij_ is the observation of j^th^ treatment and i^th^ pen; *μ* is the overall means of the sampled observation; *τ*_j_ is the effect of treatment, and *ε*_ij_ is the experimental error component.

**Table 1:** Ingredients and composition of experimental diets

**Ingredients (%) and Calculated analysis**	**Control**	**Fish oil**	**Olive oil**	**Grape seed oil**	**Canola oil**	**Soybean oil**
**Corn**	62.10	49.90	49.90	49.90	49.90	49.90
**Soybean meal**	26.90	28.71	28.71	28.71	28.71	28.71
**Barley**	-	7.12	7.12	7.12	7.12	7.12
**Calcium carbonate**	8.50	8.73	8.73	8.73	8.73	8.73
**Dicalcium phosphate**	1.53	1.55	1.55	1.55	1.55	1.55
**Salt**	0.36	0.36	0.36	0.36	0.36	0.36
**Vit premix** 1	0.25	0.25	0.25	0.25	0.25	0.25
**Min premix** 2	0.25	0.25	0.25	0.25	0.25	0.25
**DL-Methionine**	0.11	0.13	0.13	0.13	0.13	0.13
**Fish oil**	-	3.00	-	-	-	-
**Olive oil**	-	-	3.00	-	-	-
**Grape seed oil**	-	-	-	3.00	-	-
**Canola oil**	-	-	-	-	3.00	-
**Soybean oil**	-	-	-	-	-	3.00
**Metabolizable energy (kcal kg** ^-1^ **)**	2684	2760	2760	2760	2760	2760
**Crude protein **	17.18	17.72	17.72	17.72	17.72	17.72
**Crude fiber **	3.25	3.49	3.49	3.49	3.49	3.49
**Calcium **	3.66	3.75	3.75	3.75	3.75	3.75
**Available Phosphorus **	0.41	0.42	0.42	0.42	0.42	0.42
**Methionine**	0.39	0.41	0.41	0.41	0.41	0.41
**Lysine **	0.89	0.92	0.92	0.92	0.92	0.92
**Cystine**	0.30	0.29	0.29	0.29	0.29	0.29
**Tryptophan**	0.36	0.35	0.35	0.35	0.35	0.35

1 Vitamin premix provided per kg of diet: Vitamin A 7,040 IU; Vitamin D_3_ 2,000 IU; Vitamin E 8.8 IU; Vitamin K_3_ 1.76 mg; Biotin, 0.12 mg; Thiamine 1.20 mg; Riboflavin 3.20 mg; Pantothenic acid 6.40 mg; Pyridoxine 1.97 mg; Niacin 28.00 mg; Vitamin B_12_ 0.008 mg; Choline 320 mg; Folic acid 0.38 mg.

2 Mineral premix provided per kg of diet: Mn 60 mg; Fe 60 mg; Zn 51.74 mg; Cu 4.80 mg; I 0.69 mg; Se 0.16 mg.

**Table 2:** Laying hens performance in response to experimental diets

**Experimental diets**	**Egg production (%)**	**Feed intake (g per day)**	**Feed conversion ratio**	**Egg mass (g per day)**	**Egg weight (g)**
**Control**	98.06	119.61	2.06	58.07	59.21
**Fish oil**	98.51	117.41	2.03	57.83	58.70
**Olive oil**	98.51	118.31	2.06	57.36	58.22
**Grape seed oil**	96.87	117.13	2.10	55.83	57.66
**Canola oil**	99.26	121.19	2.10	57.63	58.06
**Soybean oil**	98.36	117.99	2.02	58.40	59.36
***p*** **-value**	0.295	0.955	0.813	0.568	0.736
**SEM**	0.249	0.970	0.016	0.345	0.736

## Results

**Fatty acid composition of different dietary oils. **The FA composition of the fish, olive, grape seed, canola and soybean oil is shown in Table 3. The fish oil of was a rich source of LC PUFA; n-3 and contained EPA (5.59 %) and DHA (14.20%). The fish oil had the highest amount of C14:0, C16:0, C18:0, EPA, DHA, SFA and total omega-3 FAs and the lowest amount of C18:2. Olive and canola oil were rich in C18:1 and had 69.00% and 58% of this FA, respectively. The grape seed and soybean oil were rich in C18:2 and had 68.00% and 55.50% of this FA, respectively.

**Performance. **The effects of different sources of oil on laying hen performance are summarized in Table 2. At the end of the experiment, egg production, feed intake, feed conversion ratio, egg weight and egg mass changes of the hens were not significantly affected by treatments (*p* > 0.05).

**Fatty acid composition of egg yolk. **The effects of different feed sources on egg yolk FAs composition is shown in Table 4. Fatty acids profile of the egg yolk was significantly affected by the treatments (except the C:16 and SFA). As shown in Table 4, the values of EPA, DHA and total omega-3 FAs were significantly higher (*p* < 0.01) in the egg yolk of laying hens fed fish oil compared to the eggs of other treatments.

Fish oil reduced C18:0, C18:2 (14.00% less than control group), C20:4 (66.00% less than control group), total omega-6 FAs and n-6/n-3 ratio in egg yolk in comparison with other groups. Egg yolk C18:2 and total omega-6 FAs from birds fed either grape seed oil were significantly higher than those of the other five oils.

Furthermore, canola oil supplementation enhanced the linolenic acid content of egg yolk. In this study, the highest C18:1 concentration was determined in the eggs of the hens in the birds fed with the olive and canola oil, which were rich in C18:2. Similarly, the C18:2 concentration of egg yolk was high in the groups fed soybean and grape seed oil, which are rich in C18:2. The highest C18:3 concentration of egg yolk was found in the group fed canola oil, which is also rich in C18:3.

**Table 3:** Fatty acid composition (%) of oils included in the diets of laying hens

**Fatty acids** 1 ** (%)**	**Fish oil**	**Olive oil**	**Grape seed oil**	**Canola oil**	**Soybean oil**
**C14**	5.61	0.10	0.00	0.08	0.34
**C16:0**	27.43	14.18	7.28	6.21	15.57
**C16:1**	7.97	0.89	0.07	0.17	0.00
**C18:0**	4.44	4.41	3.99	2.67	3.97
**C18:1**	32.84	68.88	20.62	57.86	23.50
**C18:2**	1.59	10.59	67.80	27.45	55.53
**C18:3**	0.23	0.84	0.24	5.56	1.10
**C20:4**	0.09	0.09	0.00	0.00	0.00
**EPA**	5.59	0.00	0.00	0.00	0.00
**DHA**	14.20	0.00	0.00	0.00	0.00
**SFA**	37.48	18.70	11.27	8.97	19.88
**MUFA**	40.81	69.77	20.69	58.02	23.50
**PUFA**	21.71	11.53	68.04	33.01	56.62
**n-3**	20.02	0.84	0.24	5.56	1.10
**n-6**	1.69	10.69	67.80	27.45	55.53
**n-6/n-3**	0.08	12.69	283.11	4.94	50.67

1 C14:0 = Myristic acid; C16:0 = Palmitic acid; C16:1 = Palmitoleic acid; C18:0 = Stearic acid; C18:1 = Oleic acid; C18:2 = Linoleic acid; C18:3 = Linolenic acid ; C20:4 = Arachidonic acid; EPA = Eicosapentenoic acid; DHA = Docosahexaenoic acid; SFA = Saturated fatty acid; MUFA = Monounsaturated fatty acid; PUFA = Polyunsaturated fatty acid; n6/n3 = The ratio of n-6 to n-3 PUFA.

**Table 4:** Fatty acid composition (%) of the egg yolk of laying hens fed different sources of oil

**Fatty acids** 1	**Control**	**Fish oil**	**Olive oil**	**Grape seed oil**	**Canola oil**	**Soybean oil**	**Significance**	**SEM**
**C14**	0.45^c^	0.51^b^	0.58^a^	0.45^bc^	0.39^d^	0.43^cd^	*	0.016
**C16:0**	29.25	29.47	29.32	30.20	27.05	26.74	NS	0.372
**C16:1**	4.47^a^	4.19^a^	3.42^b^	3.13 bc	3.16^bc^	2.90^c^	*	0.131
**C18:0**	7.86^bc^	6.95^d^	7.63^c^	8.16 b	7.58^c^	8.95^a^	*	0.134
**C18:1**	45.66^a^	45.86^a^	47.00^a^	37.06^c^	46.99^a^	41.27^b^	*	0.837
**C18:2**	10.21^d^	8.76^e^	9.79^d^	18.72^a^	12.29^c^	17.32^b^	*	0.898
**C18:3**	0.17^e^	0.28^d^	0.37^c^	0.10^f^	0.81^a^	0.55^b^	*	0.052
**C20:4**	1.74^ab^	0.59^c^	1.52^b^	1.97^a^	1.74^ab^	1.83^a^	*	0.099
**EPA**	0.00^b^	0.18^a^	0.00^b^	0.00^b^	0.00^b^	0.00^b^	*	0.014
**DHA**	0.19^c^	3.21^a^	0.37^b^	0.20^c^	0.00^d^	0.00^b^	*	0.243
**SFA**	37.56	36.93	37.53	38.82	35.01	36.13	NS	0.342
**MUFA**	50.13^a^	50.04^a^	50.42^a^	40.19^c^	50.15^a^	44.17^b^	*	0.905
**PUFA**	12.31^d^	13.02^d^	12.05^d^	20.99^a^	14.83^c^	19.70^b^	*	0.841
**n-3**	0.36^e^	3.66^a^	0.74 c	0.30^e^	0.81^b^	0.55^d^	*	0.246
**n-6**	11.95^d^	9.35^e^	11.31^d^	20.69^a^	14.03^c^	19.15^b^	*	0.965
**n-6/n-3**	33.52^b^	2.55^d^	15.33^c^	68.61^a^	17.45^c^	34.94^b^	*	4.398

a–f Different superscripts indicate significant differences (*p* < 0.05). NS: Not significant; SEM: Standard error of the mean; *: *p* < 0.01.

1 EPA = Eicosapentenoic acid; DHA = Docosahexaenoic acid; SFA = Saturated fatty acid; MUFA = Monounsaturated fatty acid; PUFA = Polyunsaturated fatty acid; n6/n3 = The ratio of n-6 to n-3 PUFA.

## Discussion

Guclu *et al.* reported that quails fed diet supplemented with sunflower and olive oil produced significantly the heaviest eggs.^15^ In another study, Kucukersan *et al*. reported that supplementation of four different kinds (sunflower, fish, soybean and hazelnut oil) of oil sources at 3.00% concentration had significant effect on egg production and egg weight.^16^ All of these results are in contrast with the present study. Also, it is indicated that supplementation of fish oil and tallow at the level of 1.50% to the corn-soybean meal diet may affect egg production performance and egg weight without any adverse effects on body weight.^17^

In present study, SFA content was not significantly different between the treatments which are in agreement with other reports.^18^^,^^19^ Hens have the ability to synthesize SFA and if the values of their rations decrease, hens can compensate the lack of these FAs.^7^

Some studies have reported that the DHA and EPA concentrations of egg yolk of birds fed diets containing fish oil is the reflection of the DHA and EPA concentration of fish oil.^7^^,^^19^^-^^23^ Reportedly, inclusion of fish oil in the diet could increase the proportion of n-3 PUFA relative to n-6 PUFA in the tissues of poultry.^24^^,^^25^

There is competition among the enzymes involved in the elongation and desaturation of omega-3 and omega-6 FAs.^26^ Delta-6 desaturase is the critical enzyme in these reactions, for which the greatest affinity appears to be conferred by the greatest number of double bonds in the C18 substrate.^27^ Hence, using the diets rich in omega-3 FAs (e.g. fish oil) reduces the omega-6 FAs contents of egg yolk.

In conclusion, the results of the present study demonstrated that different oil sources had varying effects on FA composition of egg yolk. This is reflected by the FA composition of the oils added to the diet. Based on the results, adding 3% fish oil to the laying hens diet, could increase DHA and EPA content of egg yolk with their consequent health benefits.
